# Supplementary material for: Structure of antiviral drug bulevirtide bound to hepatitis B and D virus receptor protein NTCP
Source: Nat Commun. 2024 Mar 20;15:2476. doi: 10.1038/s41467-024-46706-w (PMC10954734; doi:10.1038/s41467-024-46706-w)
Supplement: Supplementary file 1 — Supplementary Information [file 41467_2024_46706_MOESM1_ESM.pdf]

Supplementary Information

Structure of antiviral drug bulevirtide bound to hepatitis B and D virus receptor protein NTCP

Hongtao Liu[1], Dariusz Zakrzewicz[2], Kamil Nosol[1], Rossitza N. Irobalieva[1], Somnath Mukherjee[3], Rose Bang-Sørensen[1], Nora Goldmann[4,5], Sebastian Kunz[2], Lorenzo Rossi[1], Anthony A. Kossiakoff[3,*], Stephan Urban[6,7,*], Dieter Glebe[4,5,*], Joachim Geyer[2,*], & Kaspar P. Locher[1,*]

[1]Institute of Molecular Biology and Biophysics, ETH Zürich, Zürich, Switzerland

[2]Institute of Pharmacology and Toxicology, Faculty of Veterinary Medicine, Justus Liebig University Giessen, Giessen, Germany

[3]Department of Biochemistry and Molecular Biology, The University of Chicago, Chicago, IL, USA

[4]Institute of Medical Virology, National Reference Centre for Hepatitis B Viruses and Hepatitis D Viruses, Justus Liebig University Giessen, Giessen, Germany

[5]German Center for Infection Research (DZIF) - Giessen-Marburg-Langen Partner Site, Giessen, Germany

[6]Department of Infectious Diseases, Molecular Virology, Heidelberg University, Heidelberg, Germany

[7]German Center for Infection Research (DZIF) - Heidelberg Partner Site, Heidelberg, Germany

*To whom correspondence may be addressed. Email: koss@bsd.uchicago.edu, Stephan.Urban@med.uni-heidelberg.de, Dieter.Glebe@viro.med.uni-giessen.de, Joachim.M.Geyer@vetmed.uni-giessen.de, locher@mol.biol.ethz.ch

This PDF file includes:

Supplementary Tables 1-2
Supplementary Figures 1-7
Supplementary References

Supplementary Table 1. Cryo-EM data collection and processing.

**Sample**	NTCP-BLV-Fab3-Nb
PDB	8RQF
EMDB	EMD-19440
**Data collection and processing**	
Microscope	Titan Krios G4
Electron gun	FEG
Energy filter	GIF BioQuantum
Detector	Gatan K3
Voltage (kV)	300
Magnification	130,000x
Electron exposure per movie ($e^-/Å^2$)	55
Defocus range (μm)	-0.5 to -2.5
Pixel size (Å)	0.648
Numbers of micrographs	2,610
Symmetry imposed	C1
Initial particle images (no.)	1,696,766
Final particle images (no.)	128,700
Map resolution (Å) at FSC threshold of 0.143	3.41
Map sharpening *B* factor ($Å^2$)	118.0
Processing software	CryoSPARC v4.4.0
**Refinement**	
Initial model used (PDB code)	7ZYI
FSC threshold (0.143/0.5)	3.4/3.6
Model composition	
Non-hydrogen atoms	6,950
Protein residues	903
Ligands	LIG: 1
B factor ($Å^2$)	
Protein	22.96/177.66/83.01
Ligand	44.34/48.12/48.12
R. m. s. d deviations	
Bond lengths (Å)	0.003
Bond angles (°)	0.640
**Validation**	
MolProbity score	1.81
Clash score	9.09
Poor rotamers (%)	0.26
Ramachandran plot	
Favored (%)	95.30
Allowed (%)	4.70
Disallowed (%)	0.00

Supplementary Table 2. List of interactions between BLV and NTCP

BLV peptide		NTCP
No.	AA	
2	Myr-G	F128, C129, L131, G132, M133, D152, Y156, K157
3	T	D152, K153, V272
4	N	D152, K153, V154, P155, Y156, T268, I269, V272
5	L	K157, G158
6	S	P155, G158, I159, Q264, T268
7	V	G158, I161, S162
8	P	P154, G158, I159, S162, N262, Q264, L265
9	N	L31, V32, L35, N262, Q264
10	P	L35, G102, N103, S162, V166, N262, V263, M290
11	L	L31, M34, L35, I38, L104
12	G	L31, V263, Q264, M290
13	F	L27, L31, V202, S206, V263, Q264, S267, F283, P286, L287, M290
14	F	F18, D24, L27, V210, Q264, S267, F283, F284, L287
15	P	Q264, S267, T268, N271, F283
16	D	G19, D24, S28, Q264
17	H	D24, L27, S28, L31, Q264
18	Q	V32, Q264, T268
19	L	V32, L35, S162, L165, V166
20	D	-
21	P	-
22	A	-
23	F	L25, S28, V29, V32
24	G	-
25	A	G19
26	N	G19, K20, D24, L25, S28
27	S	G19, K20
28	N	K20
29	N	-
30	P	K20
31	D	-
32	W	T268, N271, V272
33	D	N271, V272
34	F	-
35	N	K153, V272, A273
36	P	-
37	N	-
38	K	N271, V272, A273, F274, P275, P276
39	D	Y146, A273, F274, P275, V278
40	H	P275, E277, V278

41	W	N87, I88, L91, I145, Y146, D147, F274, V278
42	P	-
43	E	N87, I145, D147
44	A	K86, N87, I88, V278
45	N	K86
46	K	L85, K86, N87
47	V	R84, L85, K86, N87
48	G	N87, G144, I145

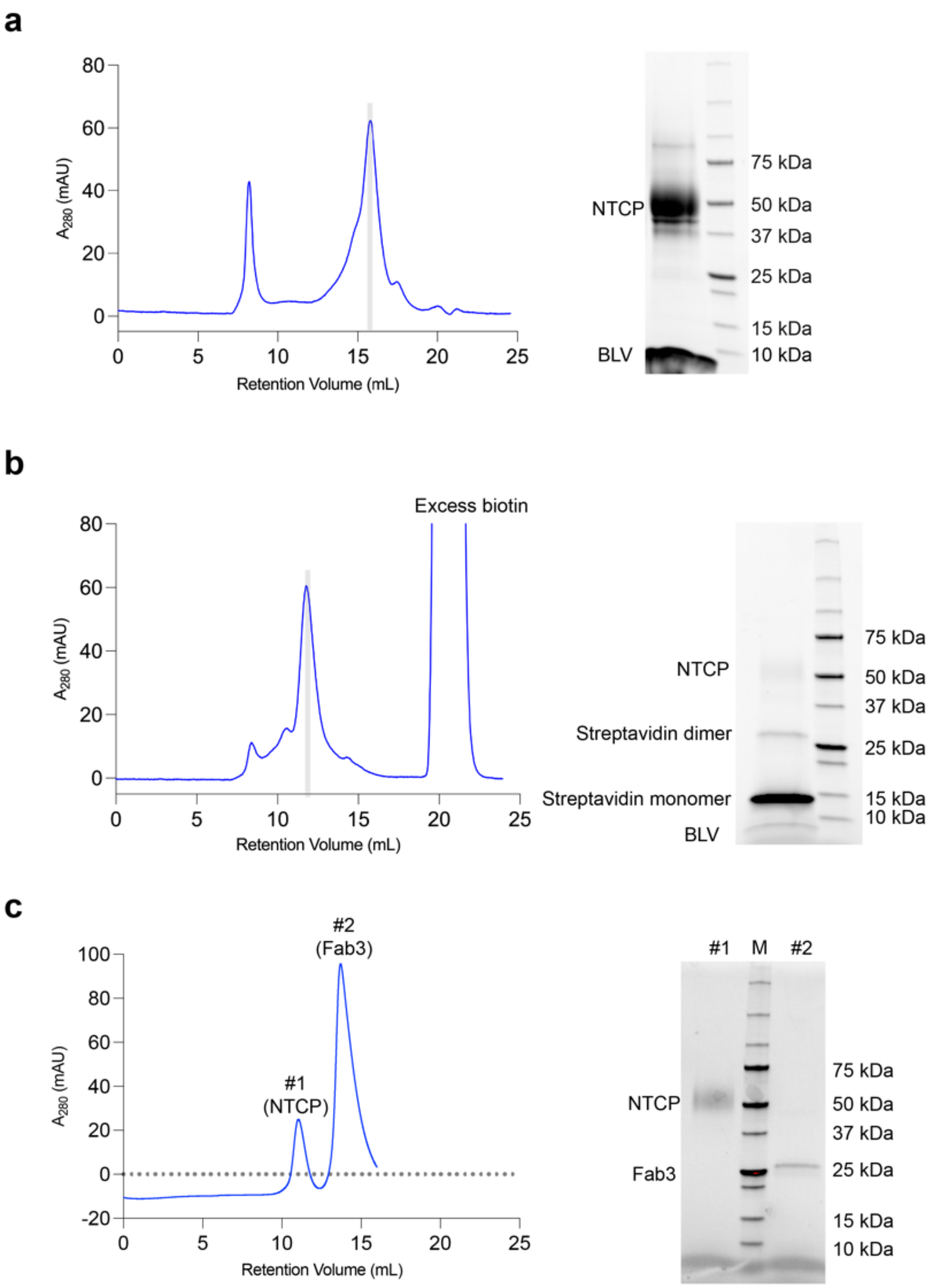

Supplementary Fig. 1. Purification of the NTCP-BLV complex.

a Left: Size-exclusion chromatogram (SEC) of detergent-solubilized NTCP bound to BLV. Right: SDS-PAGE analysis of the highlighted fraction. **b** Left: SEC of NTCP-BLV complex after biotinylation. Highlighted fraction was used for pull-down assay. Right: SDS-PAGE analysis of a sample immobilized on streptavidin-coated paramagnetic particles (see Methods section). **c** Left: SEC of NTCP and Fab3 mixture in the absence of BLV, confirming lack of complex formation. Right: SDS-PAGE analysis of selected fractions.

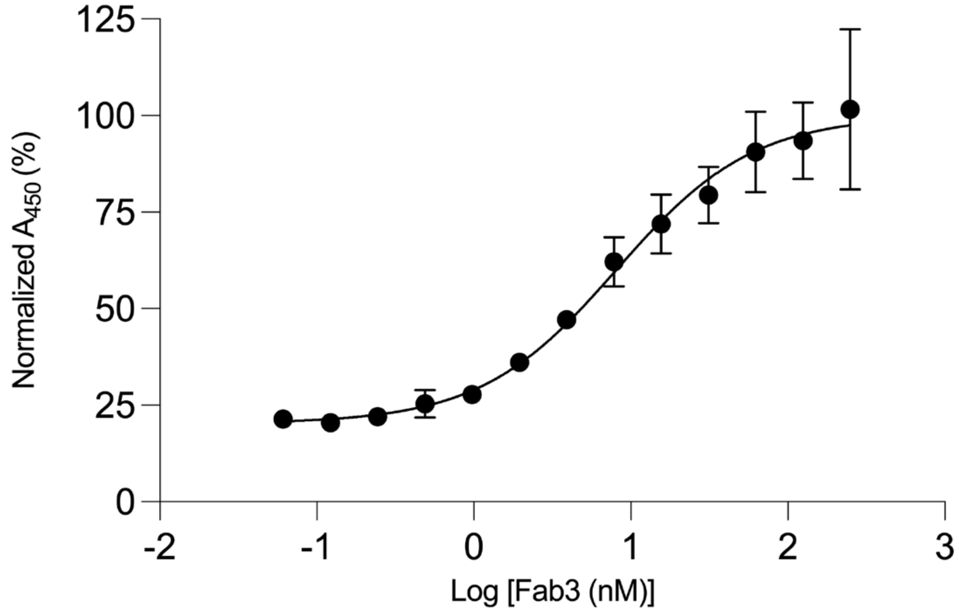

Supplementary Fig. 2. Estimation of the binding affinity (EC_{50}) of Fab3 to BLV-bound NTCP in detergent.

Normalized protein ELISA at varying concentrations of Fab3 to determine the binding affinity, EC_{50}, of Fab3 to detergent-solubilized BLV-bound NTCP. The EC_{50} was calculated to be 8 nM. Data points represent the mean, error bars indicate the standard deviation of three independent measurements.

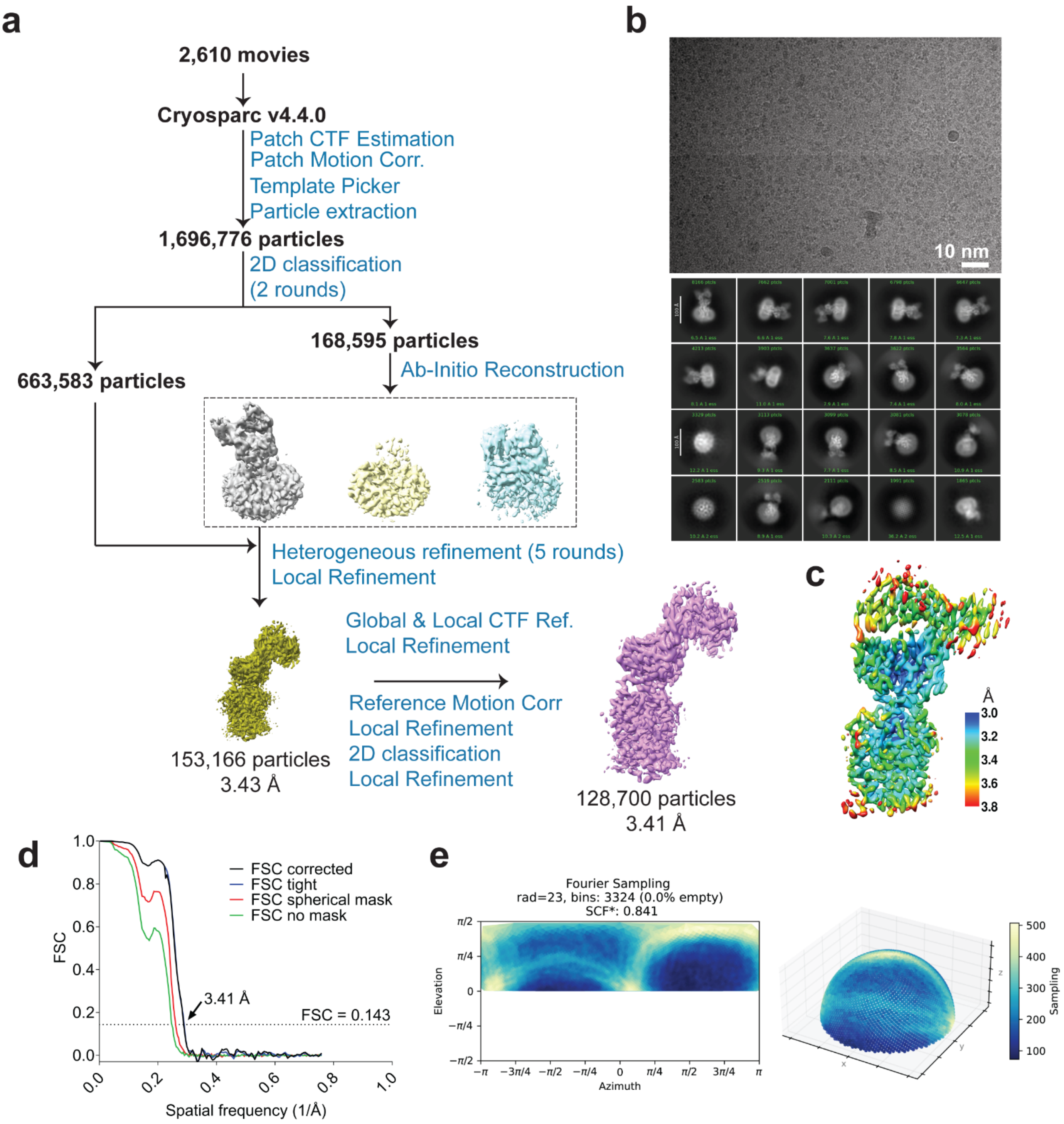

Supplementary Fig. 3. Cryo-EM data processing of the NTCP-BLV-Fab3-Nb complex.

a Data processing flowchart. **b** Representative motion-corrected micrograph and 2D class averages. **c** Final map colored by local resolution estimation. **d** Fourier shell correlation (FSC) and **e** orientation distribution as calculated in CryoSPARC. The Sampling Compensation factor (SCF) has a range [0,1] with higher values indicating better distribution[1].

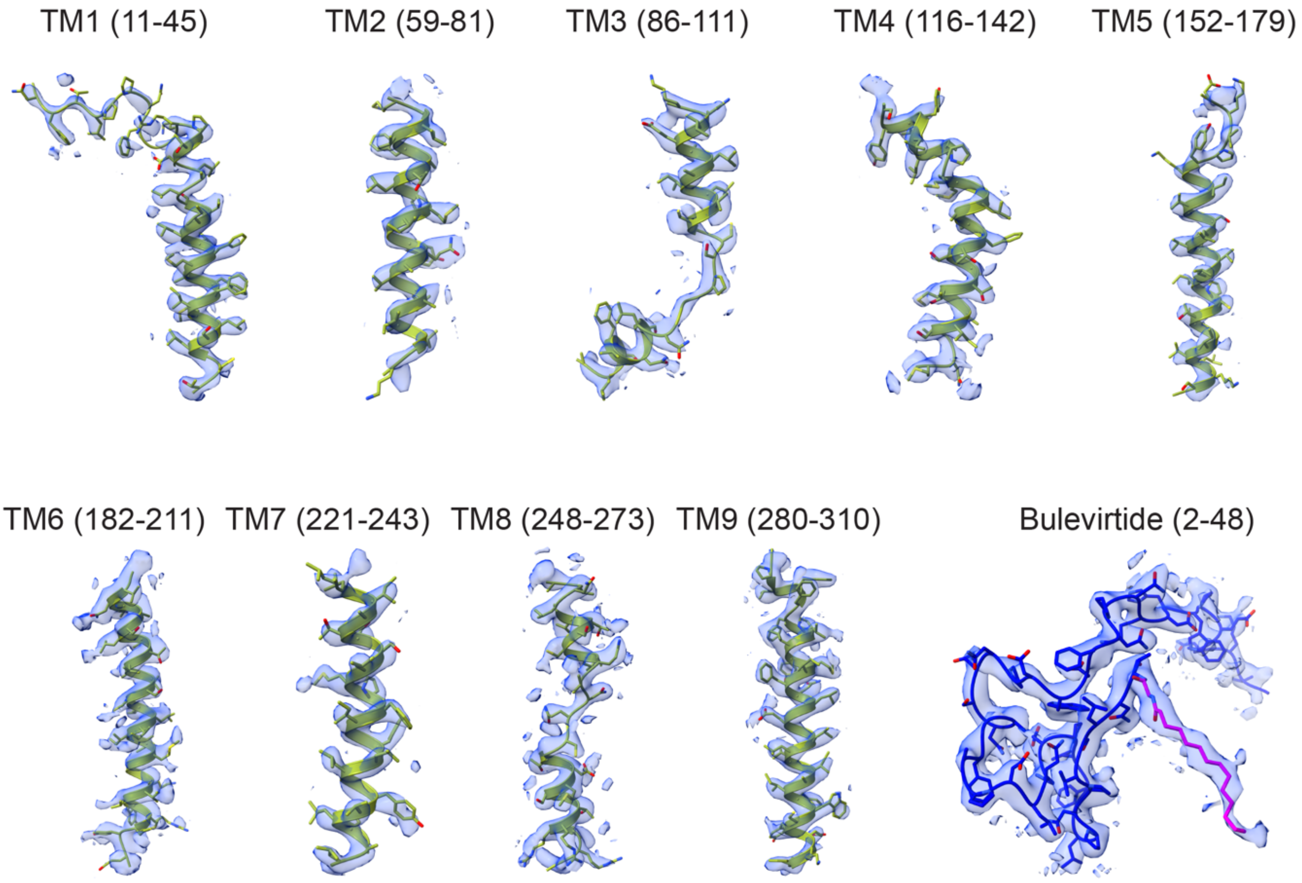

Supplementary Fig. 4. Cryo-EM densities of the individual NTCP TM helices and Bulevirtide

TM helices of NTCP are shown in green and the Bulevirtide peptide in blue. The corresponding EM density is displayed as transparent blue surface.

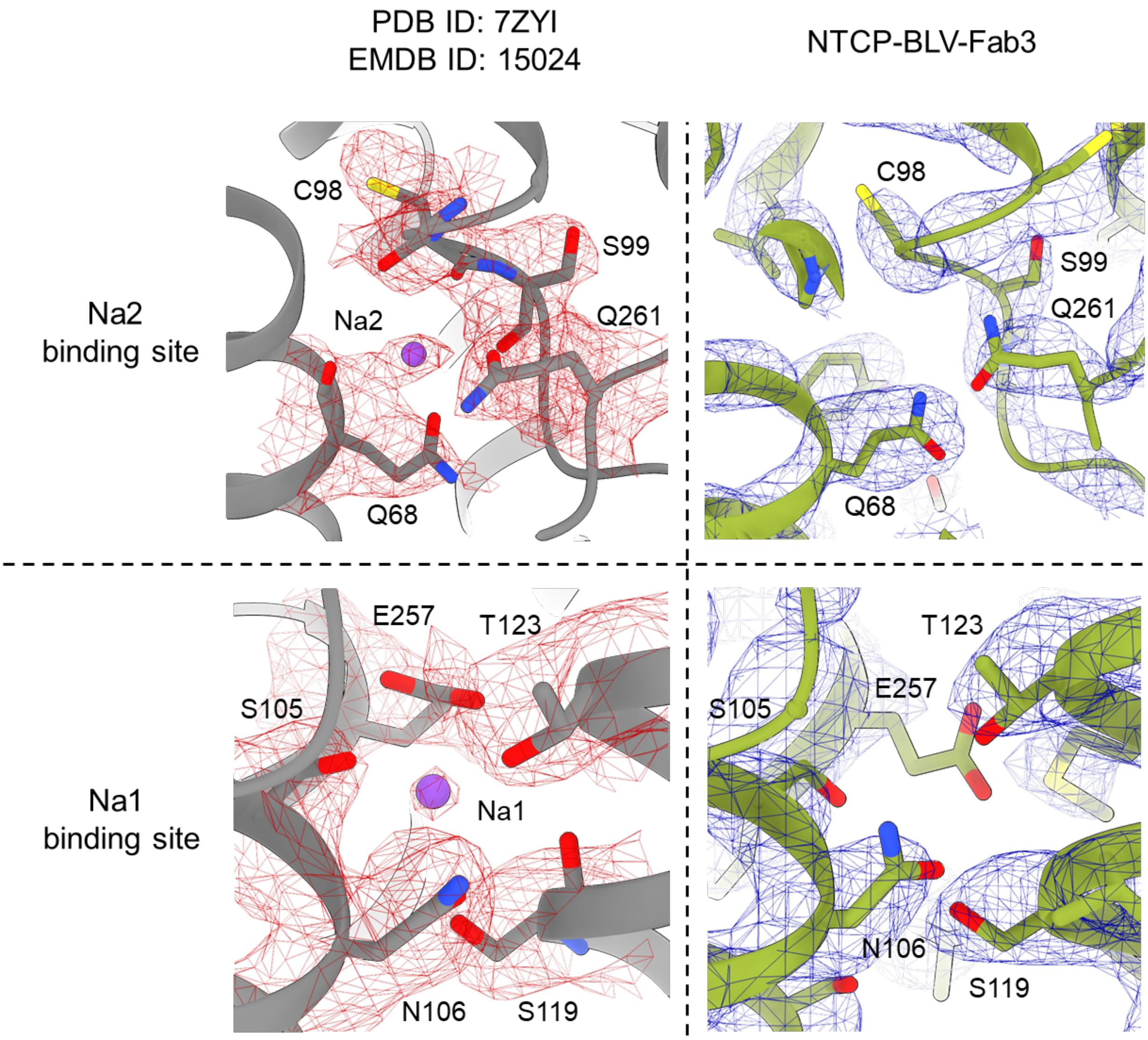

Supplementary Fig. 5. Comparison of sodium binding sites in substrate-bound and BLV-bound NTCP.

The structure of substrate-bound NTCP (PDB ID: 7ZYI, EMDB ID: 15024) is shown in grey ribbon representation, and residues coordinating bound sodium ions (Na1 and Na2) are shown in stick representation and labeled. The EM density is shown as red mesh. The structure of BLV-bound NTCP (this study) is shown in green ribbon representation, residues are labeled, and the EM density is shown as blue mesh.

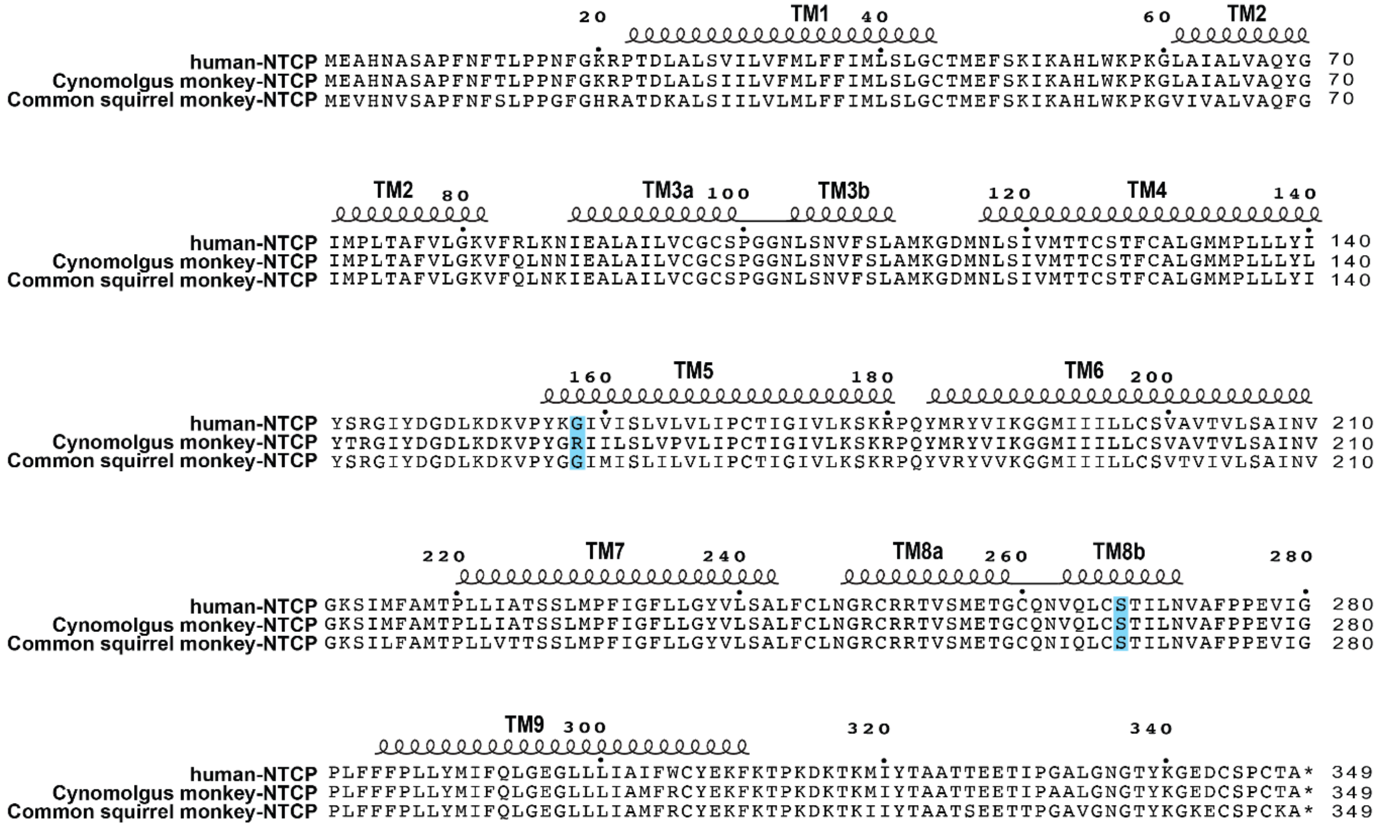

Supplementary Fig. 6. Sequence alignment of NTCP among different primates.

Amino acid sequence alignment of NTCP from human, Cynomolgus monkey (representative of Old World Monkeys), and Common squirrel monkey (representative of New World Monkeys). Residues highlighted in blue are discussed in the text.

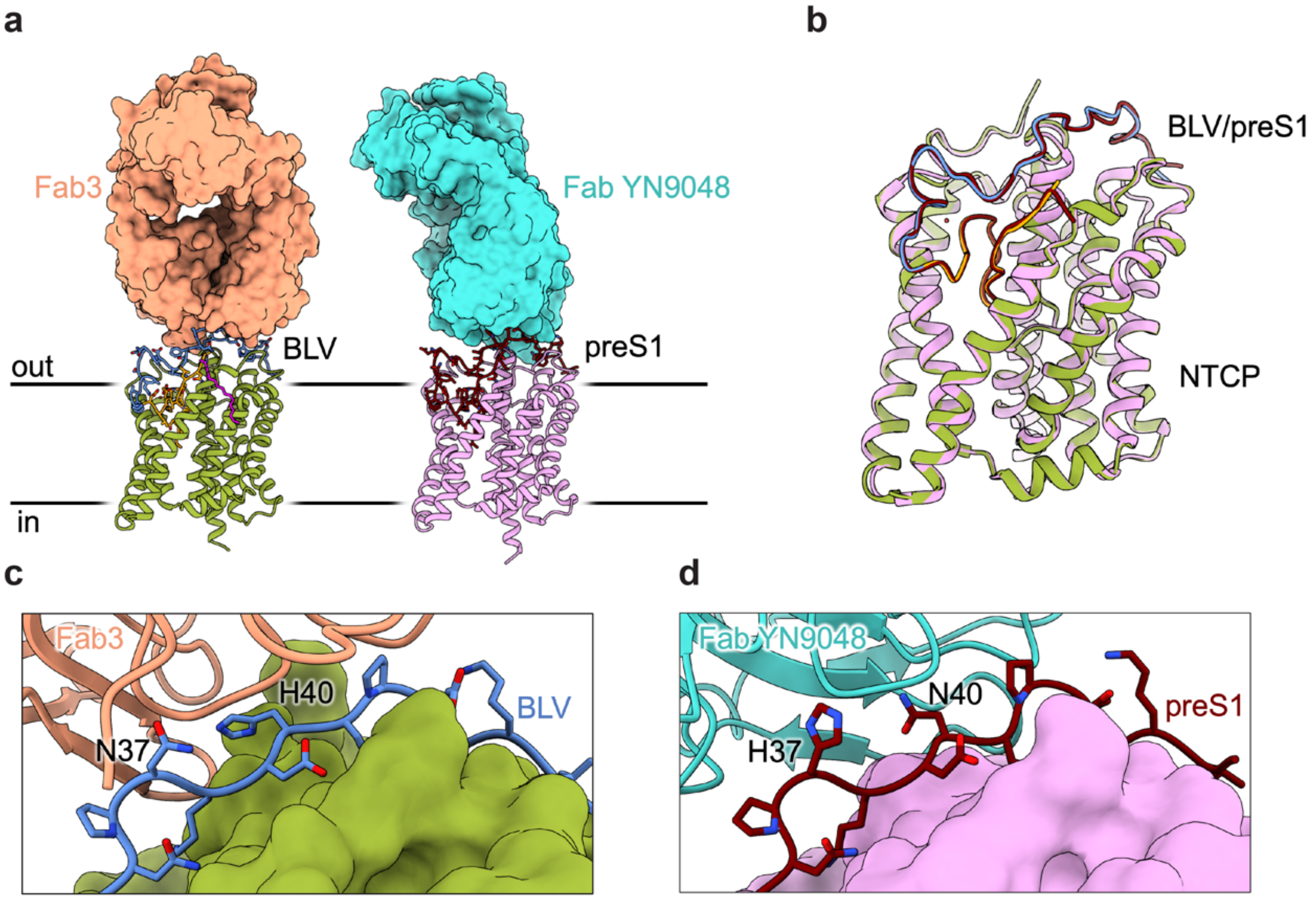

Supplementary Fig. 7. Comparison of BLV-bound and preS1-bound NTCP structures.

a Structure of NTCP-BLV-Fab3-Nb (this study) and NTCP-preS1-Fab YN9048 (PDB ID: 8HRX)[2]. NTCP is displayed in ribbon representation, Fabs as surface, and bound peptides as sticks. **b** Superposition of BLV-bound and preS1-bound NTCP structures, colored as in panel **a**. **c-d** Close-up view of the peptides interacting with the corresponding Fabs. The two residues labeled are involved in Fab binding and differ between BLV (**c**) and preS1 (**d**).

Supplementary References

1 Baldwin, P.R. and Lyumkis, D. Tools for visualizing and analyzing Fourier space sampling in Cryo-EM. *Prog. Biophys. Mol. Biol.* (2021). https://doi.org/10.1016/j.pbiomolbio.2019.09.002

2 Asami, J. *et al.* Structural basis of hepatitis B virus receptor binding. *Nat Struct Mol Biol* (2024). https://doi.org/10.1038/s41594-023-01191-5
