# Supplementary material for: Structure of antiviral drug bulevirtide bound to hepatitis B and D virus receptor protein NTCP
Source: Nat Commun. 2024 Mar 20;15:2476. doi: 10.1038/s41467-024-46706-w (PMC10954734; doi:10.1038/s41467-024-46706-w)
Supplement: Supplementary file 3 — Reporting Summary [file 41467_2024_46706_MOESM3_ESM.pdf]

nature portfolio

Corresponding author(s): Kaspar P. Locher, Joachim Geyer, Dieter Glebe, Anthony A. Kossiakoff, Stephan Urban

Last updated by author(s): Feb 23, 2024

Reporting Summary

Nature Portfolio wishes to improve the reproducibility of the work that we publish. This form provides structure for consistency and transparency in reporting. For further information on Nature Portfolio policies, see our Editorial Policies and the Editorial Policy Checklist.

Statistics

For all statistical analyses, confirm that the following items are present in the figure legend, table legend, main text, or Methods section.

n/a	Confirmed	
☐	☒	The exact sample size (*n*) for each experimental group/condition, given as a discrete number and unit of measurement
☐	☒	A statement on whether measurements were taken from distinct samples or whether the same sample was measured repeatedly
☐	☒	The statistical test(s) used AND whether they are one- or two-sided<br>*Only common tests should be described solely by name; describe more complex techniques in the Methods section.*
☒	☐	A description of all covariates tested
☐	☒	A description of any assumptions or corrections, such as tests of normality and adjustment for multiple comparisons
☐	☒	A full description of the statistical parameters including central tendency (e.g. means) or other basic estimates (e.g. regression coefficient) AND variation (e.g. standard deviation) or associated estimates of uncertainty (e.g. confidence intervals)
☐	☒	For null hypothesis testing, the test statistic (e.g. *F*, *t*, *r*) with confidence intervals, effect sizes, degrees of freedom and *P* value noted<br>*Give P values as exact values whenever suitable.*
☒	☐	For Bayesian analysis, information on the choice of priors and Markov chain Monte Carlo settings
☒	☐	For hierarchical and complex designs, identification of the appropriate level for tests and full reporting of outcomes
☒	☐	Estimates of effect sizes (e.g. Cohen's *d*, Pearson's *r*), indicating how they were calculated

Our web collection on statistics for biologists contains articles on many of the points above.

Software and code

Policy information about availability of computer code

Data collection	EPU2 software (Thermo Fisher Scientific)
Data analysis	Cryo-EM data processing: CryoSPARC4.4.0<br>Model building: Coot 0.9.8.3 (ccp4), PHENIX 1.18.2<br>Data analysis and visualisation: USCF ChimeraX 1.6.1 and UCSF Chimera 1.15, GraphPad Prism 9 and 10, ClustalOmega 1.2.2, PyMOL Molecular Graphics System v2.5 Schrödinger, ChemDraw Version 22.2

For manuscripts utilizing custom algorithms or software that are central to the research but not yet described in published literature, software must be made available to editors and reviewers. We strongly encourage code deposition in a community repository (e.g. GitHub). See the Nature Portfolio guidelines for submitting code & software for further information.

Data

Policy information about availability of data

All manuscripts must include a data availability statement. This statement should provide the following information, where applicable:

- Accession codes, unique identifiers, or web links for publicly available datasets
- A description of any restrictions on data availability
- For clinical datasets or third party data, please ensure that the statement adheres to our policy

Atomic coordinates of NTCP_BLV_Fab3_Nb have been deposited in The Protein Data Bank under accession code 8RQF. The 3D cryo-EM density post-processed map has been deposited in the Electron Microscopy Data Bank (EMDB) under accession number 19440.
Atomic coordinates of the substrate-bound NTCP structure can be found under the codes: PDB ID: 7ZYI, EMDB: 15024.
Atomic coordinates of the preS1-bound NTCP structure can be found under the code: PDB ID: 8HRX.

Research involving human participants, their data, or biological material

Policy information about studies with human participants or human data. See also policy information about sex, gender (identity/presentation), and sexual orientation and race, ethnicity and racism.

Reporting on sex and gender	not applicable
Reporting on race, ethnicity, or other socially relevant groupings	not applicable
Population characteristics	not applicable
Recruitment	not applicable
Ethics oversight	Use of human plasma samples were granted by the Ethics Committee of the Justus Liebig University Giessen, Giessen Germany (vote AZ 257/18).

Note that full information on the approval of the study protocol must also be provided in the manuscript.

Field-specific reporting

Please select the one below that is the best fit for your research. If you are not sure, read the appropriate sections before making your selection.

☒ Life sciences ☐ Behavioural & social sciences ☐ Ecological, evolutionary & environmental sciences

For a reference copy of the document with all sections, see nature.com/documents/nr-reporting-summary-flat.pdf

Life sciences study design

All studies must disclose on these points even when the disclosure is negative.

Sample size	Cell based transport assay of TC in the presence of BLV and SVP binding experiments were performed with N=3 biological replicates.<br>Cell based preS1 binding assays were performed with N=4 biological replicates.<br>Similar numbers of biological replicates have previously been shown to produce conclusive results (e.g. Liu, H. et al. Structure of human NTCP reveals the basis of recognition and sodium-driven transport of bile salts into the liver. Cell Res 32, 773–776 (2022))<br>For cryo-EM studies, 2500+ micrographs were used, which yielded a 3.41 Å structure.
Data exclusions	no data was excluded
Replication	Single particle analysis is an averaging method, and is therefore not used to perform replicates. The cell-based transport assay is reproducible, as it was performed 3 times independently yielding the same result.
Randomization	No randomization as no factors could have biased the results of the functional assays
Blinding	n/a for structural study and cell-based transport assay, as the study was performed on a single protein sample and thus blinding of the data would not be possible

Reporting for specific materials, systems and methods

We require information from authors about some types of materials, experimental systems and methods used in many studies. Here, indicate whether each material, system or method listed is relevant to your study. If you are not sure if a list item applies to your research, read the appropriate section before selecting a response.

Materials & experimental systems

n/a	Involved in the study	
☐	☒	Antibodies
☐	☒	Eukaryotic cell lines
☒	☐	Palaeontology and archaeology
☒	☐	Animals and other organisms
☒	☐	Clinical data
☒	☐	Dual use research of concern
☒	☐	Plants

Methods

n/a	Involved in the study	
☒	☐	ChIP-seq
☒	☐	Flow cytometry
☒	☐	MRI-based neuroimaging

Antibodies

Antibodies used	Produced in-house from a synthetic library: Fab3 (as described in methods)
Validation	Fab3 was biophysically and biochemically characterized (described in results)

Eukaryotic cell lines

Policy information about cell lines and Sex and Gender in Research

Cell line source(s)	Only Flp-In™ T-REx™ 293 cells were used in this study
Authentication	None performed
Mycoplasma contamination	Not tested
Commonly misidentified lines (See ICLAC register)	Not used

Plants

Seed stocks	n/a
Novel plant genotypes	n/a
Authentication	n/a
